# Using animal models to overcome temporal, spatial and combinatorial challenges in HIV persistence research

**DOI:** 10.1186/s12967-016-0807-y

**Published:** 2016-02-09

**Authors:** Paul W. Denton, Ole S. Søgaard, Martin Tolstrup

**Affiliations:** Institute of Clinical Medicine, Aarhus University, Palle Juul-Jensens Boulevard 99, 8200 Aarhus, Denmark; Department of Infectious Diseases, Aarhus University Hospital, Skejby, Aarhus, Denmark; Aarhus Institute for Advanced Studies, Aarhus University, Aarhus, Denmark

**Keywords:** HIV, Viral persistence, Latency, Humanized mice, Non-human primates, SIV, SHIV

## Abstract

Research challenges associated with understanding HIV persistence during antiretroviral therapy can be categorized as temporal, spatial and combinatorial. Temporal research challenges relate to the timing of events during establishment and maintenance of HIV persistence. Spatial research challenges regard the anatomical locations and cell subsets that harbor persistent HIV. Combinatorial research challenges pertain to the order of administration, timing of administration and specific combinations of compounds to be administered during HIV eradication therapy. Overcoming these challenges will improve our understanding of HIV persistence and move the field closer to achieving eradication of persistent HIV. Given that humanized mice and non-human primate HIV models permit rigorous control of experimental conditions, these models have been used extensively as in vivo research platforms for directly addressing these research challenges. The aim of this manuscript is to provide a comprehensive review of these recent translational advances made in animal models of HIV persistence.

## Background

**Fig. 1:**
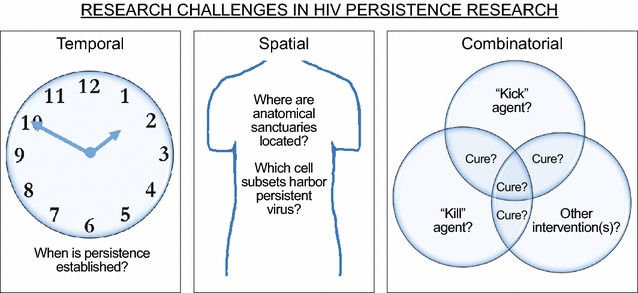
Major challenges in HIV persistence research. Categorical depictions of open questions regarding the establishment, maintenance and eradication of HIV reservoirs. The purpose of “Kick” agents is to reactivate latent virus while “kill” agents enhance the destruction of cells producing reactivated virus [129]

Key to the development of an HIV cure strategy is gaining a comprehensive understanding of the reservoir of replication-competent virus that persists despite suppressive antiretroviral therapy (ART) [1–10]. In vivo studies conducted both in humans and in animal models of HIV persistence provide critical insights regarding establishment of the reservoir, maintenance of HIV persistence and the efficacy of combination strategies for the eradication of persistent virus. Because there is inherent difficulty associated with evaluating these particular aspects of HIV persistence in clinical trials, preclinical studies in animal models of HIV disease are important guides to clinical trial designs. Reviewed here are preclinical studies in humanized mouse and non-human primate (NHP) HIV models that seek to overcome temporal, spatial or combinatorial research challenges to gain new insights into the establishment, maintenance and eradication of HIV reservoirs (Fig. 1).

## Humanized mice and NHP HIV models

Preclinical animal studies permit systematic characterizations of multiple aspects of HIV infection and viral persistence under defined conditions including demarcated infection parameters, such as time of infection, and assured compliance with therapeutic regimens. The vast majority of HIV persistence research in animal models has been performed using either humanized mice or NHP HIV models. Feline immunodeficiency virus (FIV) is the only non-primate lentivirus that causes immunodeficiency and FIV infection models have also been used in HIV persistence research [11, 12]. However, two critical challenges associated with translating FIV outcomes to the clinic (i.e., the infected cells are not primate derived and the virus is not human tropic) are overcome by humanized mouse and NHP HIV models. Consequently, this review is focused on the latter two models of HIV persistence.

**Fig. 2:**
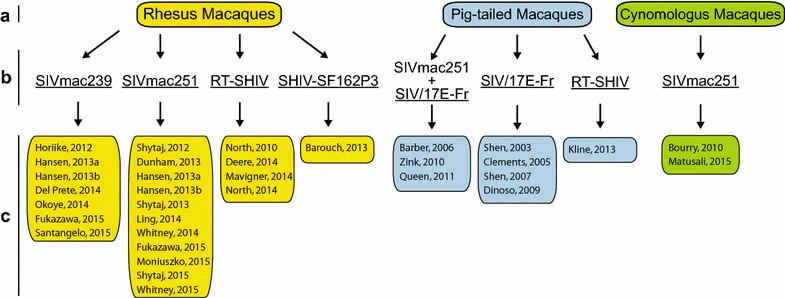
Non-human primate species and viruses utilized in HIV persistence research. NHP and SIV/SHIV combinations are indicated with references wherein the specified combination was used in the study of HIV persistence. **a** The Macaque species utilized; **b** the virus strain(s) utilized; and **c** the individual studies using the indicated combination

Under the umbrella terms “NHP” and “humanized mice” are a myriad of noteworthy model characteristics. Regarding NHP, three species have been used in HIV persistence research to date: rhesus macaques (*Macaca mulatta*), pig-tailed macaques (*M. nemestrina*) and cynomolgus macaques (*M. fascicularis*) [13–42]. These macaques were infected with simian immunodeficiency virus (SIV) or SIV/HIV chimeric viruses (SHIV) (Fig. 2). While the macaque species and immunodeficiency virus combinations may differ between studies, these NHP models all involve infecting animals that are among humans’ closest living relatives with virus that is phylogenetically linked to the human tropic virus [43]. NHP in these experiments are outbred with normal anatomy and physiology (e.g., immuno-competence); important parallels to the clinical trials that are performed in humans. For humanized mice, varieties of humanized mice and their research applications have been exhaustively reviewed previously [44–57]. The current review focuses solely on humanized mouse models utilized in HIV persistence research. Common to these models is the presence of human cells in immunodeficient mice that are infected by human-tropic virus which can be targeted by human drugs. When humanized mice are bioengineered, multiple animals with the same human genetics are created. Also, multiple human donors can be used to make cohorts of mice which allow researchers to recapitulate the diversity found in clinical trials. The general aim during the generation of humanized mice is to produce animals where a functioning human immune system is present in vivo, nevertheless the human-cell/human-virus/human-drug interactions will necessarily occur within the context of mouse anatomy and physiology during the relatively short experimental window of a mouse’s lifespan. Thus, NHP and humanized mice have defined characteristics which relate to advantages and disadvantages for each model. These characteristics must be considered when determining which experimental applications are suitable for each model. Examples of experimental applications that are essentially model specific include: (1) NHP live much longer than humanized mice which means that a persistence study that involves long-term therapy would best be conducted in a NHP system. (2) It is possible to bioengineer “personalized humanized mice” that reproduce a specific human’s immune system in multiple rodents. Experiments that require such a defined genetic background for the human immune cells can only be performed in humanized mice.

**Fig. 3:**
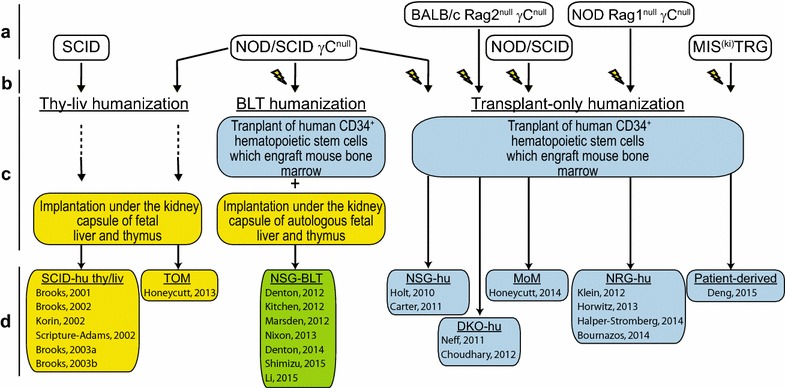
Chimerization strategies for bioengineering humanized mice utilized in HIV persistence research. **a** Immunodeficient mouse strains used for humanization in HIV persistence studies. **b** *Lightning symbols* indicate that mice received gamma radiation preconditioning. **c** The humanization strategy employed is indicated and the type of human cell and/or tissue transplanted is detailed. **d** Name associated with each type of resultant humanized mouse is listed with references wherein that type of humanized mouse was used in the study of HIV persistence

As humanized mice are individually bioengineered and not bred, some notes on the various approaches used for generating mice used in HIV persistence research are provided here. The production of humanized mice occurs during four general phases (Fig. 3). Phase A is the choosing of an immunodeficient mouse strain based on their ability to engraft human cells and tissues [44–57]. Factors that may dictate this choice include the level of immunodeficiency exhibited [58], propensity for specific tissue engraftment with human immune cells (e.g., humanization of intestinal tissues [59, 60]) and transgenic expression of human cytokines and growth factors to improve human chimerization [54]. Phase B is choosing whether to precondition the animals with gamma radiation or chemotherapy. Preconditioning becomes particularly beneficial when there is a human hematopoietic stem cell (hHSC) transplantation component to Phase C—the implantation and/or transplantation of human cells/tissues into the immunodeficient mouse. Phase D is allowing the optimal time for proper chimerization. In the case of hHSC transplantation, there is typically robust human immune system chimerization in peripheral blood by 8–12 weeks following transplant. Figure 3 highlights key variables specific to distinct humanized mice used in HIV persistence research (i.e., SCID-hu thy/liv; TOM; NSG-BLT; NSG-hu; DKO-hu; MoM; NRG-hu and Patient-derived) [61–84].

Humanized mice and NHP HIV models have been used extensively as in vivo systems for characterizing aspects of virus persistence. These studies are discussed below within the context of three categories: (1) studies that examined temporal aspects of persistence by maximizing the use of precision experimental timing (e.g., virus exposure and ART initiation); (2) studies focused on the spatial aspects of persistence where specific anatomical locations and cell types as viral reservoirs have been emphasized; and (3) studies that provided preclinical efficacy measures for interventions that could become components of a combination strategy to cure HIV.

## Temporal research challenges

The identification of infected individuals during the first days of infection requires extraordinary surveillance in communities of “at risk” individuals [85]. The difficulties associated with the assembly of a clinical trial cohort of individuals at the earliest stages of infection, combined with the fact that the eclipse phase of HIV infection lasts nearly 2 weeks [86], limit clinical studies evaluating the very earliest events in the establishment of HIV persistence to extraordinary circumstances (e.g., the case of the “Mississippi Baby” where ART was initiated in an in utero infected infant within 30 h of birth [87]). In contrast, animal models are readily amenable to precision coordination of experimental variables (e.g., timing of virus exposure, ART initiation and ART interruptions) in order to overcome temporal challenges in HIV persistence research.

Several research groups have made important observations about the earliest events involved in the establishment of the persistent HIV reservoir [26, 30, 39, 80]. Two of these groups initiated very early ART for short-term treatment following parenteral infection: Bourry et al. at 4 h (NHP) and Li et al. at 6 h (humanized mice) [39, 80]. Both groups continued the ART for 2 weeks, during which plasma viremia was very low in the NHP and remained undetected in the limited amount of blood than can be serially harvested from humanized mice. In the NHP study, the animals were harvested at the 2 week time point and multiple tissues (i.e., spleen, peripheral LN, mesenteric LN, ileum and colon) were evaluated for the presence of viral DNA and RNA. Both nucleic acid species were detected in the spleen and mesenteric LN of multiple animals that initiated ART 4 h post infection. The protocol was different in the humanized mouse study where a 3 week analytical treatment interruption (ATI) was begun following the initial 2 weeks of ART and then the animals were treated with a CD8^+^ T cell-depleting antibody every third day for ~2 months. During the ATI and CD8^+^ T cell-depletion period, some animals exhibited intermittent low level plasma viremia. Post-mortem analyses revealed the presence of viral DNA by PCR, but not viral RNA by in situ hybridization in the humanized mice that initiated ART 6 h post infection. These studies that used the initiation of ART within a few hours of infection highlight that HIV persistence is established within the first hours following infection.

A separate pair of studies examined NHP which initiated extended ART regimens within days of infection. Okoye et al. initiated ART on Day 7 post infection. Plasma viremia was reduced during the first weeks of therapy after which plasma viremia remained undetectable for the >200 days the animals were maintained on therapy [30]. This study used the quantitative viral outgrowth assay (qVOA) to quantitate the number of resting memory CD4^+^ T cells harboring latent, replication competent HIV proviruses (considered to be the greatest obstacle in the search for an HIV cure [3, 8–10]). The qVOA results are reported as infectious units per million resting memory CD4^+^ T cells (IUPM). When Okoye et al. employed this assay in their study, they failed to detect the presence of replication competent, persistent virus. This result indicates that early, prolonged ART severely limits the size of the persistent viral reservoir. However, the study by Whitney et al. showed that even when initiating ART on day 3 post infection the persistent viral reservoir does maintain the capacity to lead to rapid viremia during ATI [26]. The choice to perform an ATI after 160 days of ART rather than utilize the qVOA as an outcome measure revealed that animals treated on day 3 did exhibit viremia soon after cessation of ART even though they had not exhibited plasma viremia at any point during the study prior to the ATI. Thus, a functional relevant persistent reservoir was established within 3 days of infection, but not eliminated by 160 days of ART. Together, these studies reiterate that by the time an individual can be diagnosed as HIV positive, the persistent viral reservoir has already been established.

The qVOA utilized by Okoye et al. is generally considered to be the current best ex vivo assay for characterizing the replication competent, persistent HIV reservoir [9, 10] and this assay has been used extensively to measure the size of the latent viral reservoir in patients on long-term ART that exhibit suppressed viremia [88–97]. Yet several lines of evidence suggest that this resource intensive assay is not sufficiently sensitive and dynamic to accurately predict ATI outcomes in HIV eradication clinical trials. The translational nature of this animal study-based conclusion is confirmed by clinical data. Chun et al. reported that an individual with a profoundly low HIV burden experienced viral rebound following ATI [98]. Similar clinical outcomes were reported for the “Boston Patients” and the “Mississippi Baby” where qVOA was unable to detect the replication competent, persistent HIV reservoir and ATI were accompanied by delayed viral rebound in all three individuals [99, 100].

There are three critical challenges associated with qVOA that could explain why this assay has failed to accurately predict ATI outcomes in animal models and in patients [9, 101]. First, cell input dictates the qVOA sensitivity. This means that lower IUPM values (the goal in eradication therapy) necessitate more cells for accurate quantification. Cell yields from NHP and humanized mice are small relative to patient-derived samples which results in lower assay sensitivity in animal studies. Second, the qVOA will detect both integrated and unintegrated virus in samples where ART has not yet exceeded 6 months [9]. This time-on-ART restriction has major implications for both animal studies which have relatively short ART duration and eradication studies where qVOA cannot reliably generate baseline IUPM values for ART-naive participants or for patients experiencing viral rebound following an ATI. Third, the stimulatory culture conditions in qVOA are not suitable to reactivate all latent viral genomes present (i.e., the qVOA conservatively underestimates the size of the latent viral reservoir by ~60-fold because of the stochastic nature of virus reactivation even under maximum stimulatory conditions) [93]. The net effect of this underestimation is a potentially large, yet undefined gap between the recorded and actual IUPM values [93] that is coupled to a wide assay confidence interval (~±0.7 log_10_ IUPM) [102, 103]. Together, these mean that changes in IUPM from baseline during eradication therapy should be greater than 0.7 log_10_ before changes can be ascribed to the intervention and not to random virus reactivation events during the conduct of the assay. Gaining this level of resolution requires high cell numbers which leads back to the first challenge mentioned in this paragraph. Thus, while the qVOA may be a suitable binary marker for reduction in the size of the latent reservoir to below detection, the qVOA is unable to serve as a reliable dynamic biomarker for moderate changes in the size of the latent reservoir caused by eradication therapy. ATI is a more sensitive and informative outcome when curative strategies are being investigated.

## Spatial research challenges

The principle anatomical site examined in clinical studies is the peripheral blood because it is relatively easily accessed for clinical analyses. The intestines and lymph nodes are the next most frequently examined anatomical location in patients [93, 95, 104–107]. Importantly, each of these sites has been characterized as important in HIV persistence. Knowing that the anatomical sites examined so far harbor persistent virus provides strong rationale for the examination of additional anatomic sites which may also provide refuge for the virus. Comprehensive characterization of all putative reservoir sites is important because a clear understanding of the distinct anatomical locations, as well as the specific cell types within these locations that harbor persistent virus, is essential to ensure appropriate penetration of drugs during eradication therapy. Animal models facilitate analyses that overcome spatial challenges in HIV persistence research as they make it feasible to simultaneously examine many anatomical compartments, as well as specific cell subsets at these sites, for the presence of persistent virus. The efforts of many research groups working to overcome spatial challenges in HIV persistence research have been stratified into categories by general topic of investigation and then discussed.

### Resting memory CD4^+^ T cells as persistent virus reservoirs

A limited number of animal research studies have employed the qVOA to quantitate the latent viral reservoir. These include a pair of NHP studies and a trio of humanized mouse studies that defined the viral reservoir during ART. In the NHP studies by Shen et al. and Dinoso et al., either 2- or 3-drug ART was administered for 5–6 months during which low level plasma viremia remained detectable in most animals [17, 23]. Replication competent persistent virus was detected in cells isolated from peripheral blood and lymph nodes at very low IUPM in both studies. In the three humanized mouse studies, the qVOA was performed on resting memory CD4^+^ T cells that were pooled from multiple organ systems within the same animal [65, 67, 69]. The humanized mice utilized by Choudhary et al. and Denton et al. harbored a full complement of human hematopoietic cells while the mice used by Honeycutt et al. were reconstituted solely with human T cells [T-cell only mice (TOM)] (Fig. 3). In all 5 studies, resting memory CD4^+^ T cells harboring replication competent virus were recovered at IUPM levels analogous to patients on ART. As no animal study to date has continued ART beyond 6 months, and some of the NHP data was generated in untreated animals, the published IUPM values in these models include both stably integrated persistent virus as well as unintegrated virus [9].

### Myeloid-lineage cells as persistent virus reservoirs

While several NHP studies have focused on neurological tissues and the potential role of myeloid-lineage cells as viral reservoirs, the presence of T cells in the animals increases the challenges associated with defining the cell lineage(s) harboring persistent virus [13, 22, 27, 28]. To address this, a novel use of humanized mice has been recently described by Honeycutt et al. [77]. The mice used in this study are NOD/SCID animals that received a bone marrow transplant of hHSCs (Fig. 2) such that the animals are systemically reconstituted with human monocytes/macrophages, B cells and dendritic cells [108]. Notably, these mice lack human T cells [109]. These mice, referred to as myeloid-only mice (MoM), exhibit a general lack of detectable viral DNA in their peripheral blood cells during HIV infection. This is reversible by the administration of granulocyte colony stimulating factor (G-CSF) which leads to blood cell viral DNA becoming detectable within 6 days of administration. This finding could mean that circulating monocytes are generally not infected in this model, but mobilization of the tissue macrophages with G-CSF leads to the presence of infected cells in the blood [77]. MoM have only been presented in a conference abstract to date. The publication of comprehensive data from this model is likely to increase our understanding of the role played by the myeloid lineages in HIV persistence.

### Hematopoietic stem cells, splenocytes and thymocytes as persistent virus reservoirs

Hematopoietic stem cells are long-lived, self-renewing cells that have the potential to perpetuate integrated HIV DNA for a very long time. Carter et al. showed that hHSC infected with CXCR4-tropic HIV and then transplanted into immunodeficient mice are capable of multi-lineage engraftment of these animals [79]. Subsequently, Nixon et al. showed that CXCR4-tropic HIV can infect hHSC in vivo in humanized mice [78]. However, the bulk of HIV variants do not use CXCR4 as a co-receptor for viral entry which should limit the contribution of HSC in virus persistence. This conclusion is consistent with the observation that HIV DNA was not detected in highly purified CD34^+^ HSC from patients on long-term ART [110].

In addition to hHSC, the Zack Laboratory has also examined the role of splenocytes [71] and thymocytes [61–64, 70, 72] in HIV persistence in humanized mice. Viral persistence in splenocytes infected with a murine heat-stable antigen (HSA)-reporter virus was studied by Marsden et al. in the absence of ART because this reporter system allowed them to evaluate viral persistence in the context of ongoing virus replication in the untreated animals [71]. To identify persistently infected cells ex vivo, HSA^+^ splenocytes were immuno-depleted. The HSA^neg^ splenocytes were then stimulated in culture in the presence of raltegravir to inhibit virus spread and to limit unintegrated viral DNA from confounding the data interpretation. Culture supernatants were examined for Gag^p24^ by ELISA and it was determined that splenocytes did harbor latent HIV in vivo but the specific cell lineage(s) that harbored persistent virus were not elucidated in this study. Years earlier, this same reporter virus was used by Brooks et al. to demonstrate that thymocytes also could harbor persistent HIV [61]. The mice used in the earlier study were engrafted with a human thymus, but did not exhibit systemic human engraftment, such that the only anatomical compartment examined in this report was the thymus. A subsequent study on HIV persistence in thymocytes showed that harboring a transcriptionally silent HIV provirus did not alter cell surface phenotypes during differentiation and maturation of T cells [62]. In contrast to the humanized mouse studies, however, Shen et al. reported that thymocytes were not the source of persistent virus in a NHP model when the animals were undergoing ART [23].

Together, these studies indicate that hematopoietic stem cells, splenocytes and thymocytes all have the potential to serve as cellular reservoirs of persistent virus, although these cell types likely do not harbor the bulk of virus that persists during long-term ART in patients. In addition, the replication competence of persistent virus in these cell lineages remains an open question.

### Anatomical location of persistent virus

Multiple research groups have sought to characterize the anatomical distribution of persistent virus during ART in NHP models. Three groups performed extensive survey studies and reported on the levels of persistent virus present in tissues throughout the body [20, 21, 29, 35]. In these papers, viral DNA and RNA quantitations from lymphoid, gastrointestinal, neurological and reproductive tissues as well as other tissues from the urinary, respiratory and circulatory tracts showed that the bulk of persistent viral nucleic acids were present in lymphoid and gastrointestinal tissues during ART. In a more tissue specific study, Matusali et al. found that virus persisted at higher levels throughout the male genital tract and in the semen of adult cynomolgus macaques on ART than was observed by North et al. in juvenile rhesus macaques [21, 33]. Matusali et al. also found that virus shedding in semen during ART was reduced in some animals following ART intensification with raltegravir. Viral persistence in the central nervous system of NHP was the focus of a series of manuscripts from the Clements Laboratory [13, 22, 27, 28]. Beyond the importance of identifying persistent virus in this anatomical location, a key finding in these works was the observation that virus coding for cytotoxic T lymphocytes (CTL) escape mutations were archived in the central nervous system during ART [22]. Surprisingly, given the intense focus on the intestines as a major anatomical site harboring persistent virus [20, 21, 29, 35, 111–124], there is no published evidence from humans or animal models that replication competent, latent HIV proviruses persist in the intestines during ART.

Common to these anatomical location analyses were their reliance upon nucleic acid measures of viral persistence. The HIV DNA measures do not distinguish between defective archived virus and replication competent virus. Thus, these nucleic acid measures overestimate the amount of replication competent virus that must be targeted by any successful HIV eradication strategy [9]. The presence of HIV RNA indicates that expression has occurred recently, but neither nucleic acid determination can measure the level of replication competent, transcriptionally silent proviruses [88].

Lymphoid tissues, particularly lymph nodes, were the focus of several other virus persistence studies [17, 18, 23]. These studies reiterated the importance of lymphoid tissues as anatomical locations that harbor persistent virus during ART. Fukazawa et al. examined the architecture of lymph nodes as a means to gain mechanistic insights into the reason that viruses persist in these organs [18]. They noted that virus producing CD4^+^ T follicular helper cells in the B cell follicles were not susceptible to immune clearance. They identified B cell follicles as regions of the lymph nodes that experience very limited infiltration by CTL. CTL exclusion from B cell follicles was described in elite controller NHP although this phenotype would also be expected during ART. In addition to nucleic acid measures of viral persistence, this study included the use the qVOA to demonstrate that the persistent viral reservoirs in the lymph nodes include replication competent virus.

### Novel strategy for the non-invasive detection of persistent HIV in vivo

Santangelo et al. recently described a novel immunoPET scan technique that they were able to use for non-invasive identification of the anatomical locations with ongoing virus production during ART in NHP [41]. This study showed that cells in lymphoid tissues and the intestines continue to exhibit virus protein expression during ART which is in keeping with other studies in animal models and patient biopsy data. The key contribution of this study is its description of a technique for the detection of virus production in multiple anatomical sites simultaneously that has the potential to be refined and applied broadly in HIV cure research.

## Combinatorial research challenges

The success of ART hinges on the combination of multiple antiretroviral agents [125]. It has been suggested that an effective HIV eradication therapy will also combine multiple interventions [126–128]. Potential classes of interventions that may be included in an eradication cocktail are: HIV reactivating compounds, immune modulatory compounds and virus suppressive compounds [1–8]. Specific drug combinations as well as the order, timing and frequency of drug administration can all be validated preclinically in animal models prior to designing clinical studies. Such an approach will improve the rational design of clinical studies to increase their probability of successfully achieving trial endpoints. Most animal studies have evaluated the impact of ART plus a single intervention on virus persistence with the expectation that combinations will be evaluated in NHP or humanized mice subsequently. These studies are discussed in the following paragraphs according to topic.

### Strategies to reactivate latent virus

The concept of viral reactivation in the context of a “kick and kill” eradication strategy is being pursued aggressively in clinical trials [129] and most agents in testing for HIV therapy are being developed for or are already in clinical use for oncology interventions [104, 130–133]. Animal models are ideal in vivo experimental platforms for the efficacy evaluation of not-yet-approved pharmaceuticals for their capacity to reactivate latent viruses. In a series of papers from the Zack laboratory, humanized mice were used to generate latently infected cells which were subjected to a range of stimulatory agents ex vivo (e.g., prostratin, PMA, PHA, and cytokines) to identify T cell signaling pathways involved in latency reversal [63, 64, 70, 72]. In a separate ex vivo study, Shen et al. determined that reactivation conditions for SIV in NHP cells (from animals described in Ref. [23]) did not overlap fully with conditions used for reactivating HIV from human cells in the context of the qVOA [24]. This outcome indicates that caution should be exercised when translating latency reactivation in vivo in NHP to clinical expectations; however, similarities between NHP and human reactivation data have been recently reported. Specifically, vorinostat given to patients [104, 130, 133] and NHP [37, 38] during ART caused modest increases in cell-associated viral RNA. The similar results in both in vivo systems are encouraging, but the characterization of latency reversal efficacy in NHP and patients during more potent latency reversal therapy will provide stronger confirmation of the translational nature of the NHP to clinical outcomes in such studies.

A robust example of the utility of animal models in overcoming combinatorial research challenges is the work of Halper-Stromberg et al. in humanized mice. This group combined multiple strategies to reactivate latent virus with strategies to improve antiviral immunity and killing of infected cells [74]. A key experiment in their study was to serially dose (five times) HIV-infected humanized mice with a mix of three broadly neutralizing antibodies (i.e., 3BNC117, 10-1074 and PG16) which suppressed plasma viremia. Then the animals received three separate doses of a combination of three virus reactivating agents. The three virus inducers used in the combination were vorinostat, an HDAC inhibitor; I-BET151, a BET protein inhibitor; and anti-CTLA-4 antibody, a T cell inhibitory pathway blocker. The remarkable observation made during this experiment was: even in the absence of ART, the combinatorial eradication strategy employed prevented viral rebound in 57 % of animals. Even though the dosing and timing of dosing were not yet optimized in this study, these observations strongly suggest that combinations of viral inducers appear to be synergistic in vivo.

### Strategies to improve antiviral immunity and the killing of infected cells

Active immunization, as well as passive immunization, are strategies being investigated for improving antiviral immunity during virus eradication therapy. Hansen et al. performed an active immunization of NHP using a cytomegalovirus (CMV) vector expressing SIV proteins [19]. This study generated excitement because long-term control of viremia was observed in the vaccine arm of the study. Amazingly, a cohort of eight vaccinated animals ceased to exhibit any plasma viremia by ~70 days post immunization and remained viremia free until necropsy (>160 days post immunization). This effect has been attributed to atypical CD8^+^ T cell targeting by the CMV vector [42]. At harvest the qVOA was utilized to demonstrate that no replication competent virus could be recovered from multiple tissues in the immunized animals. This outcome is very encouraging and the hurdles to implementing this strategy in clinical trials are being addressed.

Passive immunization with broadly neutralizing antibodies has been evaluated for its therapeutic potential in humanized mouse and NHP HIV models. The antibodies were administered either in combination with [75] or in the absence of [16, 73, 76] ART. In all cases, significant reductions in viremia were associated with antibody administration. Furthermore, broadly neutralizing antibodies combined with ART also led to a reduction in cell-associated viral DNA. Immunotoxins are a form of passive immunization where Fab fragments of antibodies are fused with a toxin to deliver the deadly payload to virus expressing cells [134]. Brooks et al. showed that an immunotoxin that specifically targets HIV envelope-expressing cells kills virus expressing cells following latency reversal ex vivo [63]. Subsequently, Denton et al. used this immunotoxin to reduce viral persistence in vivo. The immunotoxin was administered to humanized mice over a 2 week period in conjunction with continued ART [68]. The outcome of this relatively short immunotoxin therapy in vivo was an additional 1 log_10_ reduction in tissue viral RNA levels beyond the impact of ART alone.

In addition to immunization, immunomodulatory strategies also have the potential to boost antiviral immunity against cells where latent virus has recently been reactivated. When toll-like receptors (TLR) recognize distinct foreign molecular patterns, they activate innate and adaptive immune cells. Whitney et al. recently published a conference report describing the effect of a TLR7 agonist given to ART-suppressed NHP [31]. In their study, they observed both reduced viral DNA in blood and tissues and a marked induction of plasma viremia following multiple oral administrations of the inhibitor. Furthermore, the viral set point in these animals was lower during ATI versus animals that did not receive the TLR7 agonist. The surprising outcome of this study suggests that it may be possible for a single molecule to function as both the “kick” and the “kill” component of a virus eradication strategy. At the least, such a TLR agonist could augment the reactivation of latent virus while simultaneously functioning as an immune enhancer.

Recently, Deng et al. showed that it is not sufficient to simply boost antiviral immunity, especially in patients who initiated ART during chronic infection [66]. The experimental conditions were highly controlled in this study as three key experimental components were independently derived from a single human donor who initiated ART during chronic infection. The three components were: (1) virus grown out from the latent reservoir using the qVOA; (2) ex vivo stimulated CD8^+^ T cells; and (3) humanized mice generated from bone marrow-derived HSC. The humanized mice were infected with virus from the patient’s latent viral reservoir and then received an adoptive transfer of the ex vivo stimulated CD8^+^ T cells. The key variable was the reagent used for the ex vivo CD8^+^ T cell stimulation. When the stimulation included immune-dominant gag-epitopes based upon the patient’s HLA genotype, the adoptively transferred cells were unable to control viremia in the recipient humanized mice. However, when the stimulation did not include epitopes for which escape mutations were pre-existing, the adoptively transferred cells were capable of controlling viremia in the recipient humanized mice. This study yielded two very valuable contributions. First, when ART is initiated during chronic infection, the latent virus reservoir harbors CTL escape variants. Second, vaccination strategies in patients may need to be tailored for individual HLA genotypes to avoid stimulating ineffective antiviral immunity.

### Strategies to suppress virus activity

Different strategies to suppress residual virus replication during ART have been tested in animal models. North et al. evaluated ART intensification regimens in NHP [32]. Animals received either 3-, 4-, or 5-drug ART regimens, but no major differences in viral decay kinetics were observed and all animals experienced viral rebound during ATI. Using a complicated delivery strategy, Shytaj et al. combined ART intensification in NHP with auranofin and buthionine sulfoximine [14, 15, 25]. The net effect of this regimen was a substantial alteration in the natural course of infection that appeared to limit disease progression to AIDS, but did not limit viral rebound during ATI. Dunham et al. coupled an indoleamine 2,3-dioxygenase inhibitor with ART in NHP, but did not observe any alteration of the inflammatory environment in vivo, as hypothesized, nor did they observe an impact on persistent infection [36]. Moniuszko et al. performed a similar study with glucocorticoid to reduce the number of proinflammatory CD16^+^ monocytes present in NHP on ART [40]. This regimen did reduce the target cell population, but did not alter the persistent viral DNA levels relative to ART alone. Mavigner et al. performed HSC transplants in NHP during ART using autologous cells to determine the impact of myeloablative total body irradiation plus stem cell transplant on viral persistence [34]. One to two months following the transplant, ART was discontinued in the experimental and control arms of the study. Rapid viral rebound and similar tissue viral nucleic levels characterized in the animals from both groups at necropsy indicated that neither the act of conditioning for a stem cell transplant nor the transplant itself were sufficient to significantly impact viral persistence. Holt et al., Neff et al., Kitchen et al. and Shimizu et al. employed various gene editing strategies to successfully reduce viral burden by reducing the number of human T cells susceptible to viral entry in HIV infected humanized mice in the absence of ART [81–84]. While these pioneering studies did not achieve the ultimate therapeutic goal of virus eradication, they confirm the feasibility of performing preclinical combinatorial trials in animal models. Regarding future animal model studies in HIV persistence, it is noteworthy that high costs and animal availability are major concerns for researchers conducting HIV persistence studies in animal models—particularly for investigators utilizing NHP. The creation of a core facility that supplies ART-suppressed animals to HIV persistence researchers could address these concerns, accelerate the work and lower the costs associated with these lines of research.

## Conclusions

Given that experimental conditions are more readily controlled in animal studies, humanized mice and NHP are ideal experimental platforms for overcoming temporal, spatial and combinatorial challenges in HIV persistence research. Importantly, since certain studies such as patient-specific humanized mice and long-term ART administration in NHP [26, 66] can only be performed in the respective model utilized, these in vivo HIV models have complementing advantages for HIV persistence researchers. Beyond model specific contributions to the field of HIV persistence research, both humanized mice and NHP models have been used independently to generate correlative data sets regarding viral persistence in anatomical regions outside the typically assayed primary and secondary lymphoid tissues. Both types of models have been used to show that the persistent viral reservoir is established within the very first hours and days of infection and early ART is insufficient to eradicate the persistent virus. Both types of models have also been used to perform preclinical efficacy evaluations of novel combinations of eradication interventions which will likely be required to achieve a cure for this disease. Both humanized mice and NHP HIV models should be utilized to evaluate potential biomarkers of viral rebound following ATI (e.g., PD-1, TIM-3, and LAG-3 [135]) as the validation of such markers will simplify clinical cure trial designs. The generation of these and other translational data in animal HIV models will continue to move the field closer to a cure for HIV disease.

## Abbreviations

ART: antiretroviral therapy
ATI: analytical treatment interruption
BALB: Bagg’s albino
BLT: bone marrow, liver, thymus
CMV: cytomegalovirus
CTL: cytotoxic T lymphocytes
DKO: double knockout
FIV: feline immunodeficiency virus
γC: common gamma chain
G-CSF: granulocyte colony stimulating factor
HSA: heat-stable antigen
IUPM: infectious units per million resting memory CD4^+^ T cells
MIS^(ki)^TRG mouse: Rag2^null^IL2rg^null^ 129xBalb/c (N2) with knock-in replacements of the endogenous mouse Csf1, Csf2, IL3, SIRP-α and Tpo with humanized versions
MoM: myeloid-cell only mice
NHP: non-human primate
NOD: non-obese diabetic
NSG: NOD/SCID-gamma chain knockout
NRG: NOD-RAG-gamma chain knockout
PHA: phytohemagglutinin
PMA: phorbol myristate acetate
qVOA: quantitative viral outgrowth assay
Rag: recombination-activating gene
RT: reverse transcriptase
SCID: severe combined immunodeficient
SHIV: simian/human chimeric immunodeficiency virus
SIV: simian immunodeficiency virus
TLR: toll-like receptor
TOM: T cell only mice
